# Feasibility of HIV point-of-care tests for resource-limited settings: challenges and solutions

**DOI:** 10.1186/s12916-014-0173-7

**Published:** 2014-09-08

**Authors:** Wendy Stevens, Natasha Gous, Nathan Ford, Lesley E Scott

**Affiliations:** Department of Molecular Medicine and Haematology, Faculty of Health Sciences, University of the Witwatersrand, Johannesburg, Gauteng South Africa; National Health Laboratory Service and National Priority Program, Johannesburg, South Africa; Department of HIV/AIDS, World Health Organization, Geneva, Switzerland

**Keywords:** Anti-retroviral therapy, CD4, Challenges, HIV, Implementation, Point-of-care, Viral load

## Abstract

Improved access to anti-retroviral therapy increases the need for affordable monitoring using assays such as CD4 and/or viral load in resource-limited settings. Barriers to accessing treatment, high rates of loss to initiation and poor retention in care are prompting the need to find alternatives to conventional centralized laboratory testing in certain countries. Strong advocacy has led to a rapidly expanding repertoire of point-of-care tests for HIV. point-of-care testing is not without its challenges: poor regulatory control, lack of guidelines, absence of quality monitoring and lack of industry standards for connectivity, to name a few. The management of HIV increasingly requires a multidisciplinary testing approach involving hematology, chemistry, and tests associated with the management of non-communicable diseases, thus added expertise is needed. This is further complicated by additional human resource requirements and the need for continuous training, a sustainable supply chain, and reimbursement strategies. It is clear that to ensure appropriate national implementation either in a tiered laboratory model or a total decentralized model, clear country-specific assessments need to be conducted.

## Introduction

Globally, the number of persons living with HIV has increased from 34 million (31.4 to 35.9 million) in 2011 to an estimated 35.3 million (32.2 to 38.8 million) in 2012; approximately 69% of the global HIV burden resides in sub-Saharan Africa [1]. In response to anti-retroviral therapy (ART) programs, a concurrent drop in AIDS-related deaths from 2.3 million (2.1 to 2.6 million) in 2005 to 1.6 million (1.4 to 1.9 million) in 2012 has been recorded [1]. In order to reach the expected 2020 goals, a massive increase in HIV testing capacity will be required.

The expansion of ART programs can only be described as a huge success in low- and middle- income countries. Estimates reached 9.7 million on ART at the end of 2012, representing some 60% of those in need at that time [2]. With the new World Health Organization (WHO) guidelines changing the CD4 test threshold for treatment initiation from mid-2013, the number of individuals infected with HIV potentially requiring access to treatment has increased to an estimated 28.6 million [1]. Challenges to continued ART scale-up remain, and include improving access to HIV testing, ensuring universal access to testing, earlier initiation of treatment by improved access to HIV testing, ensuring subsequent linkage to care, and finally long-term retention in care. Each phase of HIV diagnosis and monitoring is supported by a number of tests conducted according to different algorithms in many high-burden countries, each with human and technical resource requirements. HIV rapid tests, used in adults in serial or parallel algorithms using one to three different assays, have been instrumental in ensuring wide-scale diagnosis and access to care, albeit with ongoing challenges to ensure quality. A recent estimate from President's Emergency Plan for AIDS Relief (PEPFAR) countries suggests over 80 million HIV rapid assays were performed in 2013 and that 11% of all assays were conducted as point-of-care tests (POCTs) (Jason Williams, personal communication).

CD4 testing has been the gatekeeper for assessing immune status and establishing eligibility for treatment and care. Treatment eligibility threshold levels have changed over time from 200 cells/μl in 2002 [3] to 350 cells/μl in 2010 [4]. More recently, the new consolidated WHO recommendations suggest initiation at CD4 counts of <500 cells/μl [5]. Further suggestions of universal access and test and treat strategies are also being evaluated and hotly debated [6]. The latter approach is already occurring for certain high-risk population groups such as those co-infected with tuberculosis (TB), pregnant women, and children under 5 years of age. CD4 count has also been used for regular monitoring of immunological recovery on treatment, generally at six-monthly intervals. CD4 testing can be done at different tiers of the laboratory service [7] and the frequent delay in linking this assay to the initiation of patient care can result in significant loss to follow-up [8]. CD4 testing is also recommended by WHO and used in South Africa as a benchmark for establishing the risk of cryptococcal infection, where testing for cryptococcal antigen can now be done at point-of-care (POC) and the onset of meningitis can be prevented if treated with fluconazole [9].

The HIV viral load (VL) assay, a nucleic-acid-based test, is used to monitor response to treatment; an undetectable viral load defines treatment success. VL testing is frequently done in centralized facilities and currently requires expensive instrumentation, technical skill, and has relatively high costs per assay. Despite these challenges, this assay has gained its rightful place in guidelines and clinical practice and is thought to be the most reliable marker for treatment success [10,11]. The development pipeline of POC VL assays promises to deliver a number of options to improve access and facilitate earlier identification of treatment failure. This will allow clinicians to avoid premature switching of regimens, particularly in regions with limited drug availability, potentially improving patient adherence and reducing the development of drug resistance [12]. Also, the percentage of failures using this assay can provide a monitor of both individual and program success [13]. As access to VL testing is improving, the role of CD4 measurements is being reassessed. Numerous studies have demonstrated that for the vast majority of people living with HIV who are receiving ART and are virally suppressed, CD4 cell count does not decline over time [14]. Other studies have shown that one third of individuals whose CD4 count was greater than 350 cells/μl had viral loads greater than 100,000 HIV RNA copies/ml [15]. A meta-analysis of seven studies assessing the accuracy of clinical or CD4 tests in predicting virological failure found a poor sensitivity of 26.6% and a positive predictive value of 49.4% [11]. This suggests that in situations where viral load is available routinely, CD4 monitoring can be reduced in frequency or stopped altogether. Recognizing this opportunity to save resources, the South African ART guidelines in 2013 recommended stopping routine CD4 monitoring in people who are stable on ART and a number of other countries are considering moving in this direction [16].

In addition to the core assays described in individuals with HIV, there are also hematology and biochemistry assays that remain important, including hemoglobin, creatinine (especially for tenofovir initiation) and liver transaminase tests as well as assays for the diagnosis of opportunistic infections such as TB and cryptococcal infection. The diagnosis and treatment of TB is critical in low- and middle- income countries where a significant proportion of individuals with HIV infection are co-infected with TB. In South Africa as an example, co-infection rates are as high as 65% to 70% [17].

To address all the needs described above and in the face of the successes of rapid tests such as those for HIV, malaria and, more recently, cryptococcal antigen, there is a drive now towards using POCT for the non-communicable diseases such as diabetes, cardiovascular disease and cancer, many of which are associated with long-term management of people living with HIV. Thus, there is an expanded list of multidisciplinary testing needs at primary health clinics (PHC). Performing and interpretation of these tests will potentially require significantly more expertise than a single rapid HIV antibody test.

### History of point-of-care testing

POCT is an old approach to testing that has been around for decades and remains as controversial today as it was when first introduced. POCT refers to testing that is performed near to or at the site of patient care, with the result leading to a possible or immediate change to patient care [18]. The rationale is largely based on a need for shortening the time to decision making. The literature provides a myriad of different definitions such as the Clinical Laboratory Standards Institute in the USA, which defines the purpose of POCT being the provision of timely results that clinically and cost-effectively contribute to management decisions [19]. The first references to POCT date back to the early 1990s and focused largely on glucose testing for diabetes and blood gas analyzers in ICUs and operating theaters [20]. The controversy around managerial, quality and regulatory ownership remains a problem and it has been suggested that this is still a ‘work in progress’ [21]. Despite this, POCT is the fastest growing segment of the diagnostic industry (10% to 14% annually), accounting for one in four tests within the developing world [22,23]. A recent review reported that POCT accounts for 25% of total laboratory revenue [24]. New diagnostics into which POCT has expanded include cardiac markers, coagulation assays, substance abuse and home-based HIV testing, to name a few [25]. Interestingly, POC devices include not only *ex vitro* but also *in vitro* and *in vivo* methodologies (continuous monitoring devices) [26]. Technological advances such as microfluidics [27], miniaturization [28], microfabrication, simple power and affordable light sources, electromagnetic actuation of fluids using microelectronics and, more recently, nanodiagnostics [29,30] have facilitated the development of more complex assays capable of placement at the POC [28]. Thus, rapid tests described for HIV diagnosis have been described as first-generation POC assays, involving antigens and antibodies and simple biochemistry and hematology; the second generation is infinitely more complex and based on cell detection or nucleic acid amplification and detection; the third generation will involve complex analyzers that could have multiplexing capabilities [31].

### Global perspective on point-of-care testing

The unmet laboratory needs for assays to address communicable diseases such as HIV, TB and malaria appear to have assisted in catalyzing the POC diagnostics industry as a whole. Both communicable and non-communicable diseases will in future reap the benefits as appropriate implementation strategies are developed [31]. This is particularly important when predictions for the future suggest that diabetes may well be a more important risk factor for TB than HIV. Global market assessments have suggested that the increase in diabetes and thus glucose testing comprises at least 10% of the global POCT market [32]. The growth in POC HIV testing has been further reinforced by strong advocacy from groups such as the WHO (One pillar of Treatment v2.0 {WHO Department of HIV/AIDS, 2011 #99}guideline [33], WHO 2013 treatment guidelines [34]), UNITAID (market catalysts; Geneva), the Bill and Melinda Gates Foundation, the Clinton Foundation, PEPFAR and the African Society of Laboratory Medicine, who have been tasked with promoting guidance and implementation in field sites. This drive has begun to address many of the factors mentioned above, such as the absence of laboratories or access to assays such as CD4 and VL testing for the diagnosis and monitoring of HIV in remote sites. Alternatives to conventional centralized testing are being driven by the high rates of loss to initiation for both HIV and TB, as well poor retention in care [35]. These activities have catalyzed funders, suppliers, users and patients in galvanizing the POC diagnostics industry into action. In addition, POCT has been incorporated into the Global Health Strategy on HIV/AIDS [36]. Both the WHO and the London School of Tropical Medicine and Hygiene have been tasked with bringing forward multi-center laboratory-based validations of POC assays followed by an evaluation of their implementation in the field [37]. Strong emphasis has also been placed on the need for monitoring the impact and cost of the interventions across the entire continuum of care. By nature of the low throughput of these technologies and the additional human resources required in the field for testing and maintenance, the total assay costs can be as expensive, or more expensive, than laboratory testing. A strong emphasis needs to be placed on innovative strategies to ensure quality for tests that are being conducted in volumes far beyond that covered by conventional laboratory quality assurance plans and accreditation status. In South Africa, there is an ISO standard (ISO22789) that has been implemented for accredited laboratories to follow if they are conducting and supporting POC testing [38]. Perhaps a similar approach to accreditation of clinic sites conducting POC testing with a simpler standard and checklist could be used to ensure quality is maintained in field-testing sites.

### The pipeline for HIV diagnosis and monitoring

There is an ever-expanding pipeline associated with the strong advocacy for POCT from global players, who maintain that universal access for HIV and TB care requires the use of POCT for earlier testing and improved retention in care. Cited advantages of POCT include improved turnaround time, greater accessibility, potentially improved patient retention and possible reduction in overall healthcare costs. However, despite the rapid growth and interest in POCT, many aspects remain controversial, in part because this approach challenges the conventional approach to laboratory testing, which remains the prevailing paradigm in many countries. In addition, while numerous early or near market entry products are available, at the time of writing few could be purchased on a large scale, outside of rapid HIV and malaria strip-based tests, and a monopoly exists of one or two suppliers with a proven track record for CD4 testing, such as the PIMA assay (Alere Inc., Waltham, MA, USA). In the VL arena, many early market entry products are available and development has been heavily funded, yet only three were available for clinical validation as of April 2014 - the LIAT™ Analyzer (IQuum, Inc., Roche) [39], Alere™ q HIV-1/2 Detect (Alere) [40] and Samba (Diagnostics for the Real-World, Ltd.) [41] - and manufacturing track records for scale-up were not available. The upcoming pipeline for HIV CD4 and VL testing with their performance characteristics are summarized in the landscape document produced annually by UNITAID [12]. A plethora of fast followers are in various stages of research, development and evaluation and include the MBio POC CD4 (MBio Diagnostics, Inc)(Co,USA) [42], Daktari CD4 Counter (Daktari Diagnostics, Inc.)(MA, USA), FACSPresto™ (BD Biosciences)(NJ, USA) [43], Visitect® (Omega Diagnostics), Zyomyx CD4 (Zyomyx, Inc.) and EMD Millipore® Muse™ (Merck)(Darmstadt,Germany) [12]. For VL testing, these include the GeneXpert® Viral Load system (Cepheid, Sweden), the EOSCAPE-HIV™ Rapid RNA Assay system (Wave 80 Biosciences)(CA,USA) [44], TrueLab™ Real Time micro PCR system (MolBio Diagnostics, Ltd.), Goa, India Savanna VL test and platform (Northwestern Global Health Foundation in collaboration with Quidel Corporation) and Bioluminescent Assay in Real Time technology (Lumora, Ltd.)(cambridgeshire,UK) [45], amongst others [12].

In countries where significant laboratory infrastructure currently exists in both the public and private sectors, the sheer volumes of testing may make total decentralization prohibitive in terms of instrumentation and human resource requirements. In these instances, POC assays may and do have a role to play where gaps in service are noted, which can be identified by approaches such as geographic information systems mapping to ensure a national ‘total coverage model’. The total coverage model is a new term being used in laboratory testing circles which refers to a tiered implementation model that includes both POC testing and different tiers of laboratory testing to ensure access for the entire national population. POCTs are also used heavily in specific niche areas such as hemoglobin in emergency rooms or renal clinics. A particular niche for the VL assay could, for example, be in maternity wards and antenatal care clinics where pregnant women infected with HIV could be monitored for risk of transmission and success of treatment strategies, and exposed infants could be tested at birth for HIV and then treatment initiated as soon as possible.

Major issues surrounding the implementation of POCT exist and include poor regulatory control, difficulties in ongoing monitoring of quality, and limited availability of guideline documents for the safe implementation of POC devices. In addition, there are few studies that report data on full economic costing for POC [46], which is likely to vary depending on tests used, diseases investigated and model input parameters.

There is a dearth of well-designed randomized controlled clinical trials (RCTs) to evaluate the outcomes and impact of the implementation of POCT. Most notable for their contributions to the POC literature are a group led by Shephard in Australia [47,48]. Although evaluating other assays in a general practitioner setting in Australia, the final study conclusions were that POCT was not inferior to laboratory-based testing, but came at a substantially higher cost that needs to be weighed against overall health benefits. Various clinical experiences were presented at a recent forum held in South Africa, with a number of studies reporting progress in RCT studies such as the Home-based Care Plus trial in Kwazulu-Natal, Rapid Initiation of Anti-retrovirals in Pregnancy (RAP) study in Cape Town, the Grand Challenges Canada RCT, and RapIT (Midrand PHC, South Africa). Results are still awaited eagerly and will help form policy but have shown clearly that POCT is just one step in a multi-step process along the continuum of care [49]. Other experiences show that POCT has great potential for certain high-risk populations such as migrants or adolescents where loss to follow-up is high and where immediate results would add value [49].

Pilot studies on the implementation of PIMA CD4 POC testing in South Africa and Mozambique have demonstrated that time to initiation is reduced; however, challenges were identified in that nurses perceived POC implementation as additional workload, and patients migrated from facilities before staff were able to track, record and file the results in patients’ folders [49]. Experiences from Mozambique showed that after the introduction of POC CD4, the loss to follow-up before CD4 staging dropped, ART initiation rate increased, and time to ART initiation was reduced from 48 days to 20 days [50]. Retention rates in care, however, remained the same. It was recommended by this group that deploying POC should be done in in conjunction with conventional testing as part of a total laboratory network and there was acknowledgement that POC testing is far from error-proof. Only 20% of Mozambique’s CD4 counts are conducted at POC. High invalid rates were noted using POC CD4 tests in this study. The authors warned that simple implementation is not always efficient - access does not necessarily mean that the patient gets care (approximately 25% of patients did not get CD4 testing even with POCT on site). They also highlighted that significant health systems strengthening is needed and clinic workflow re-engineering. A meta-analysis of the performance of PIMA is underway and preliminary analysis have revealed that the performance of the instrument on venous specimens is as good as current gold standard technology. However, the performance on capillary-derived specimens showed increased variability at the 350 cells/μl threshold, resulting in higher false-positive rates that would lead to more patients being placed on ART (unpublished results, Lesley Scott personal communication).

### Approaches to ensuring quality testing

The US Food and Drug Administration requirements for defining a simple test are that it should be rapid, easy to perform, require minimal training and no specialized laboratory setup, and reagents should be stable and temperature independent. However, few assays actually meet these requirements. It should be noted that assay transfer from the laboratory to POC is not synonymous with improved quality of care. Implementation at the POC will require facilitation in a step-wise fashion with careful monitoring and evaluation at each step. The approach to quality of rapid lateral flow-based assays will be different to those that are device based. Several guidelines for HIV rapid testing have been written over the years, but uptake of these recommendations has been poor in most resource-limited settings. In fact, many of these assays are considered Clinical Laboratory Improvement Amendment-waived because they are simple tests with a low risk for an incorrect result and are thus not quality assured in developed countries such as the USA.

While programs such as the WHO pre-qualification process [51] have provided guidance by conducting product and supplier evaluations and validations, and the Centers for Disease Control and Prevention (CDC) has done similar work for PEPFAR-related programs, there is a need for harmonization of approaches and standardization of protocols with greater co-operation between stakeholders. There needs to be co-ordination between and a review of all strategies and guidelines so that a simple, single guidance can be provided for countries. Quality needs to be addressed, within the laboratory and at the pre-analytic, analytical and post-analytical phases [22]. For rapid assays, the sheer volumes of assays conducted make conventional internal and external quality approaches extremely difficult to implement. Strategies employed have included the use of external quality assurance (EQA) material using dried tube spots for various HIV rapid assays [52] or dried culture spots for near POCT for TB [53,54]. Innovative strategies are required for material distribution and data collection across large programs. Regular training and re-training, competency assessments, and ongoing supervision and mentoring of staff conducting assays are all critical to ensuring continuous maintenance of quality.

For device-based assays, an approach that is under scrutiny is the use of real-time continuous monitoring using various connectivity systems linked to analyzers in the field [55,56]. Connectivity provides a means not only to ensure analyzer performance meets requirements, but also of collecting programmatic data, distribution of results and identifying the need to intervene should problems arise. Data ownership and data security are issues that need to be addressed. Each analyzer, however, frequently connects to the middleware or software solution via a different mechanism and there is thus a need for industry standards for POCT connectivity [57]. Several middleware programs have been evaluated that link to laboratory information systems in South Africa with success, although approaches differ in different regions depending on wireless availability, internet access and computer literacy. Thus solutions may need to be contextualized within different geographic regions. Simpler approaches may include the use of bi-directional short message service printers with additional capabilities for data collection and acknowledgement of receipt of results [58]. To improve retention in care, patients can be recalled for results, and this makes for a reasonably successful means of improving adherence [59]. The role of secondary and tertiary laboratories in the management of quality in PHCs is essential and many believe POCT should be a natural extension of the laboratory [60].

Supply chain management and procurement strategies need to be well planned. Global procurement and global forecasting may play a larger role than for other assays because the production lines for new assays entering the market are frequently unable to meet the demand of rapid recommendations that lead to rapid global uptake. Engagement with industry in the pre-market phase may help to ensure quality features are built into the system, connectivity is considered, and production meets the needs based on information provided on disease prevalence and likely test numbers. UNITAID, as an organization that funds approaches to catalyze and effect market changes, can stimulate additional approaches improving access. Advocacy for quality assured, appropriately selected assays used in settings where impact can be demonstrated is strongly needed.

### Ownership and accountability

There is a general consensus that ownership should be at the level of in-country ministries of health. A POCT policy needs to be embedded within national strategic laboratory plans, the development of which was strongly advocated for by the Maputo declaration [61]. A single strategic national plan for the introduction of POCT in a country is likely to solicit donor funding or that of local treasuries in a far more effective manner. It is imperative that technical task teams are established to support decision making. The composition of the team should include clinicians; laboratorians; health economists; procurement, supply and distribution workers; and funders. Strong partnerships with industry need to be facilitated because the ongoing procurement, maintenance of analyzers and product failures need to be addressed. As a result of recent product failures in the HIV arena impacting many countries, a task team was established with expertise from organizations such as the WHO, CDC and other partners. This may be useful going forward to urgently address product failures as this body is formalized. This brings in the concept of a far more active reporting to support post-market surveillance, currently poorly coordinated the world over. Ownership of the POCT process, however, needs to extend to users of the assays and the communities that are tested, with creative ways developed for incentivizing healthcare workers conducting the tests to maintain high quality standards.

## Conclusions

POCT will improve access to needed HIV and associated diagnostics, but these assays are not without limitations that should be noted and reported. There is a need to integrate these technologies cost-effectively and efficiently into clinical algorithms and existing laboratory networks. In costing, it should be emphasized that context matters, particularly human resources and test volumes. There is much to be done in this field. Notably, large randomized studies measuring the impact of a diagnostic intervention along the entire continuum of care are currently an exception and need to be encouraged and supported. Standardization of assay evaluation and development of appropriate internal and external quality control are important activities that need support. Regulatory hurdles need to be overcome and developed in many countries. Global harmonization of all stakeholder activities is essential to get the product from an idea to the bench and ultimately to the patient bedside. The likelihood is that in many countries POCT will be strategically deployed in a hybrid model with support from the conventional tiers of in-country laboratories.

## Abbreviations

ART: antiretroviral therapy
CDC: Centers for Disease Control and Prevention
EQA: external quality assurance
PEPFAR: President’s Emergency Plan for AIDS Relief
PHC: primary health-care clinic
POC: point-of-care
POCT: point-of-care testing
RCT: randomized control trial
TB: tuberculosis
VL: viral load
WHO: World Health Oganization
